# Seeing Beyond the Tract: The Superiority of 3 Tesla (3T) MRI Over X-ray Fistulography in Perianal Fistula Evaluation

**DOI:** 10.7759/cureus.93825

**Published:** 2025-10-04

**Authors:** Sachin Khanduri, Mohammad Shahbaz, Sana, Archiya Habib, Somya Singhal, Akshay Aggarwal, Nisha, Avani Kanojia, Kunal N Dwari, Saleem Khan

**Affiliations:** 1 Radiodiagnosis, Era's Lucknow Medical College and Hospital, Lucknow, IND

**Keywords:** 3 tesla mri, fistula-in-ano, perianal fistula, preoperative imaging, st. james classification, x-ray fistulography

## Abstract

Background

Perianal fistulas are a prevalent anorectal condition, often resulting from cryptoglandular infections. They cause significant morbidity due to chronic discharge, pain, and recurrent infections, affecting patient's quality of life. Accurate preoperative imaging is crucial for defining the fistulous tract, identifying internal and external openings, and planning surgical intervention to prevent recurrence. X-ray fistulography is outdated due to its poor visualization of soft tissues and inability to adequately image complex anatomical structures, making it a less preferred method in modern clinical practice. High spatial resolution magnetic resonance imaging (MRI), especially using 3 Tesla (3T) scanners, has emerged as the preferred modality due to its excellent soft tissue characterization and multiplanar imaging capability.

Aim

This study aims to evaluate and compare the diagnostic efficacy of 3T MRI fistulogram and conventional X-ray fistulography in patients with clinically suspected perianal fistulas and to correlate imaging findings with intraoperative surgical observations.

Materials and methods

A prospective cross-sectional study was conducted over 24 months in a tertiary care center in Lucknow. A total of 52 patients with clinical suspicion of perianal fistulas were enrolled. All patients underwent both X-ray fistulography and 3T MRI fistulography prior to surgical management. Imaging findings were evaluated for tract length, width, complexity, internal and external openings, and secondary extensions. MRI-based classification was performed using the St. James University Hospital (SJUH) grading system. Intraoperative findings served as the reference standard. Data were statistically analyzed to assess agreement and diagnostic accuracy.

Results

The mean age of patients was 37.4 ± 11.03 years, with a male predominance (92.3%). All patients presented with perianal pus discharge; 55.8% also reported pain. MRI showed significantly longer (mean: 6.21 ± 0.93 cm) and wider (mean: 3.59 ± 0.43 mm) tracts compared to X-ray (length: 5.50 ± 1.10 cm; width: 3.13 ± 0.53 mm), with p < 0.01. MRI more accurately detected internal and external openings and complex tracts. According to the SJUH system, 76.9% were complex fistulas, with 55.8% graded as grade 4. MRI findings had high agreement with surgical findings (κ = 0.820). The sensitivity and specificity of MRI for fistula detection were both 100%. For detecting high-grade fistulas (grades 4 and 5), MRI showed a sensitivity of 96.6% and specificity of 93.9%. X-ray fistulography frequently missed secondary tracts and internal openings and showed poor agreement with surgical outcomes.

Conclusion

3T MRI fistulography is a highly accurate and superior imaging modality for preoperative assessment of perianal fistulas. It offers a detailed visualization of tract morphology and strong concordance with intraoperative findings, significantly outperforming conventional X-ray fistulography. MRI is expected to remain the imaging gold standard for the evaluation of fistula-in-ano, whereas the role of X-ray fistulography is anticipated to further decline given its limited diagnostic value.

## Introduction

Perianal fistulas are debilitating anorectal conditions that significantly impair a patient’s quality of life. They represent abnormal epithelialized tracts connecting the anal canal to the perianal skin, most commonly arising from unresolved cryptoglandular infections. Clinically, they present with symptoms such as pain, purulent discharge, pruritus ani, and, in some cases, fecal incontinence. Epidemiological studies indicate a higher prevalence in males, with an incidence of 12.3 per 100,000 in men compared to 5.6 per 100,000 in women [1]. The mean age at diagnosis is approximately 38 years, with peak occurrence between the second and fourth decades of life [2]. Risk factors include obesity, diabetes mellitus, smoking, dyslipidemia, and physical inactivity. Additionally, chronic inflammatory conditions such as Crohn’s disease and infections like tuberculosis may also contribute to fistula formation [3].

Anatomically, fistulas are classified into intersphincteric, transsphincteric, suprasphincteric, and extrasphincteric types, based on their course in relation to the anal sphincter complex. Precise preoperative imaging is vital for identifying the primary tract, internal opening, secondary extensions, and sphincter involvement, all of which are essential for optimal surgical planning and reducing the risk of recurrence.

Historically, X-ray fistulography, performed by instilling contrast into the fistulous tract, was used to delineate fistula anatomy. However, its clinical value has progressively declined due to inherent limitations, including invasiveness, suboptimal soft-tissue contrast, incomplete assessment of complex tracts, and risk of contrast-related complications [4]. Similarly, plain radiographs, although occasionally helpful for detecting bony involvement or associated abscesses, provide limited information on soft tissue anatomy.

Endoanal or transrectal ultrasound (EUS/TRUS) offers high-resolution visualization of the anal sphincter and surrounding structures and can be enhanced by 3D imaging or hydrogen peroxide instillation. However, its diagnostic utility is constrained by operator dependence and limited sensitivity in detecting high-lying or complex fistulas [5].

Magnetic resonance imaging (MRI) has emerged as the gold standard in the evaluation of perianal fistulas, owing to its superior soft tissue contrast, multiplanar imaging capabilities, and noninvasive nature [6]. MRI enables a comprehensive assessment of the fistulous tract, including internal openings, secondary ramifications, and adjacent inflammatory changes. High-field 3 Tesla (3T) MRI systems, in combination with surface coils and advanced techniques such as diffusion-weighted imaging (DWI) and post-contrast imaging, further enhance diagnostic precision. Routine MRI protocols incorporate T1-weighted, T2-weighted, fat-suppressed, and post-contrast sequences, often acquired in oblique planes aligned to the anal canal to improve anatomical delineation.

Given the limitations of traditional imaging modalities and the diagnostic advantages of MRI, the present study aims to compare the diagnostic efficacy of 3T MRI fistulogram with conventional X-ray fistulogram. Additionally, it seeks to correlate imaging findings with intraoperative observations, thereby underscoring the clinical utility and anatomical accuracy of MRI, particularly in the evaluation of complex perianal fistulas.

## Materials and methods

This cross-sectional study was conducted over a period of 24 months at the Department of Radiodiagnosis, in collaboration with the Department of Surgery, at a tertiary care center in Lucknow. A total of 52 patients with clinically suspected perianal fistulas and scheduled for surgical intervention (fistulotomy or fistulectomy) were prospectively enrolled after obtaining informed written consent. Each patient underwent both X-ray fistulogram and MRI fistulogram prior to surgery.

Patients presenting with purulent discharge from the perianal region and clinical suspicion of a perianal fistula were included in the study. Exclusion criteria comprised the presence of contraindications to MRI, such as ferromagnetic pacemakers, metallic implants, anal stenosis, or claustrophobia. Patients who were on antibiotic or anti-inflammatory therapy during the week of enrollment, as well as those who declined surgical management, were also excluded.

Demographic and clinical details were recorded for each participant. MRI was performed using a 3T scanner with standard T1-weighted, T2-weighted, and DWI sequences. Image interpretation and post-processing were carried out using Syngovia software by an experienced radiologist blinded to the clinical and surgical findings.

All imaging findings were documented, including fistula length, width, tract classification, and identification of internal and external openings. The final diagnosis was confirmed intraoperatively and served as the reference standard for comparison. Data were compiled using MS Excel (Microsoft Corporation, Redmond, Washington, United States) and analyzed using appropriate statistical tests to compare MRI and X-ray findings, with a p-value of less than 0.05 considered statistically significant.

## Results

A total of 52 patients with clinically suspected perianal fistulas who were scheduled for surgical intervention were enrolled in the study. The age of participants ranged from 19 to 67 years, with a mean age of 37.40 ± 11.03 years. The highest proportion of patients (38.5%) belonged to the 31-40-year age group. The cohort showed a marked male predominance, with 92.3% of patients being male. Most patients (63.5%) reported symptoms persisting for 16 to 30 days, with a mean symptom duration of 20.52 ± 7.48 days. All patients presented with purulent perianal discharge, and 55.8% also complained of pain. A previous surgical history was noted in 15.4% of patients.

**Figure 1:**
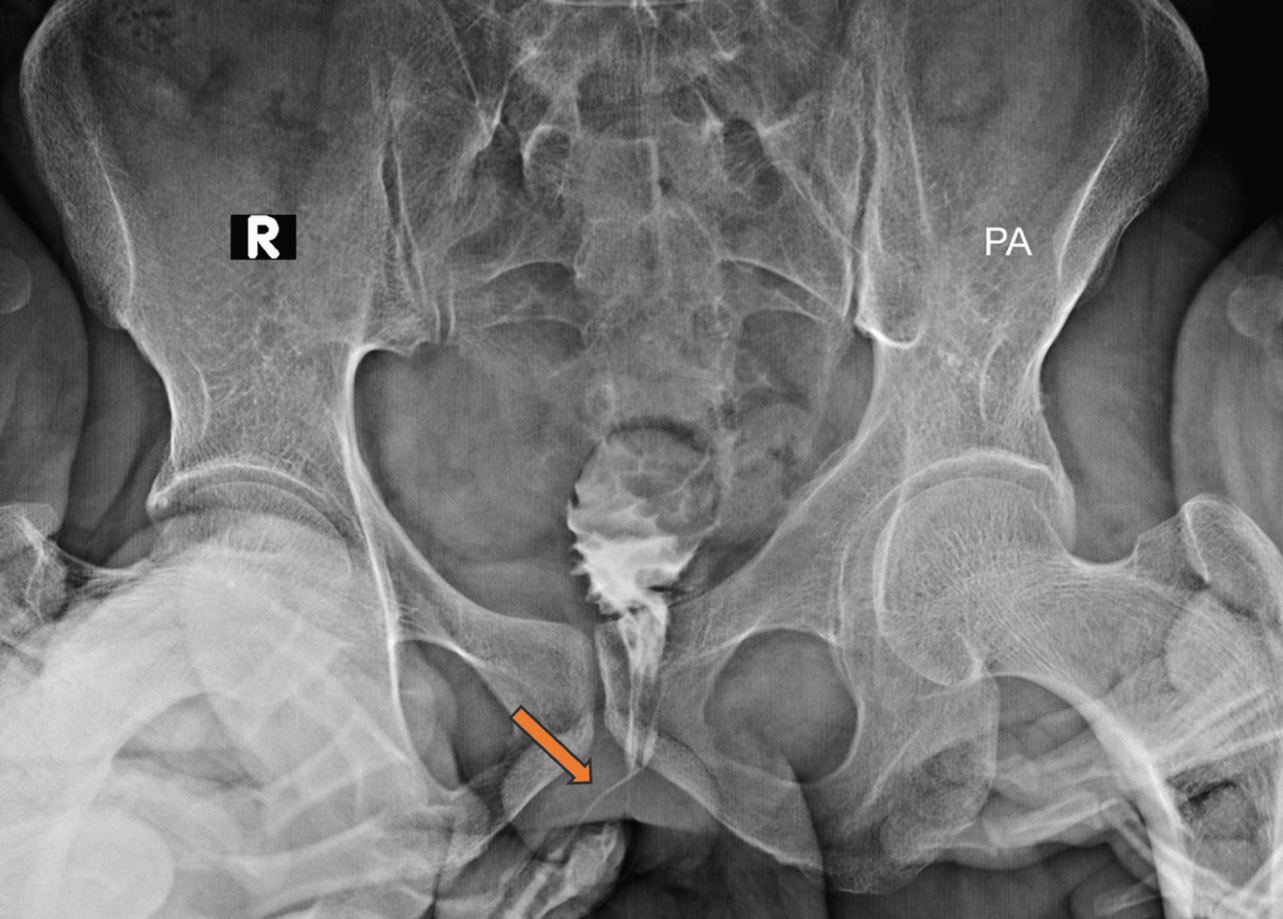
X-ray fistulogram showing contrast-filled perianal fistulous tract in PA view (orange arrow). The tract is outlined from the external opening toward the anal canal. Limited soft tissue detail highlights the modality’s constraints compared to MRI PA: posteroanterior; MRI: magnetic resonance imaging

**Figure 2:**
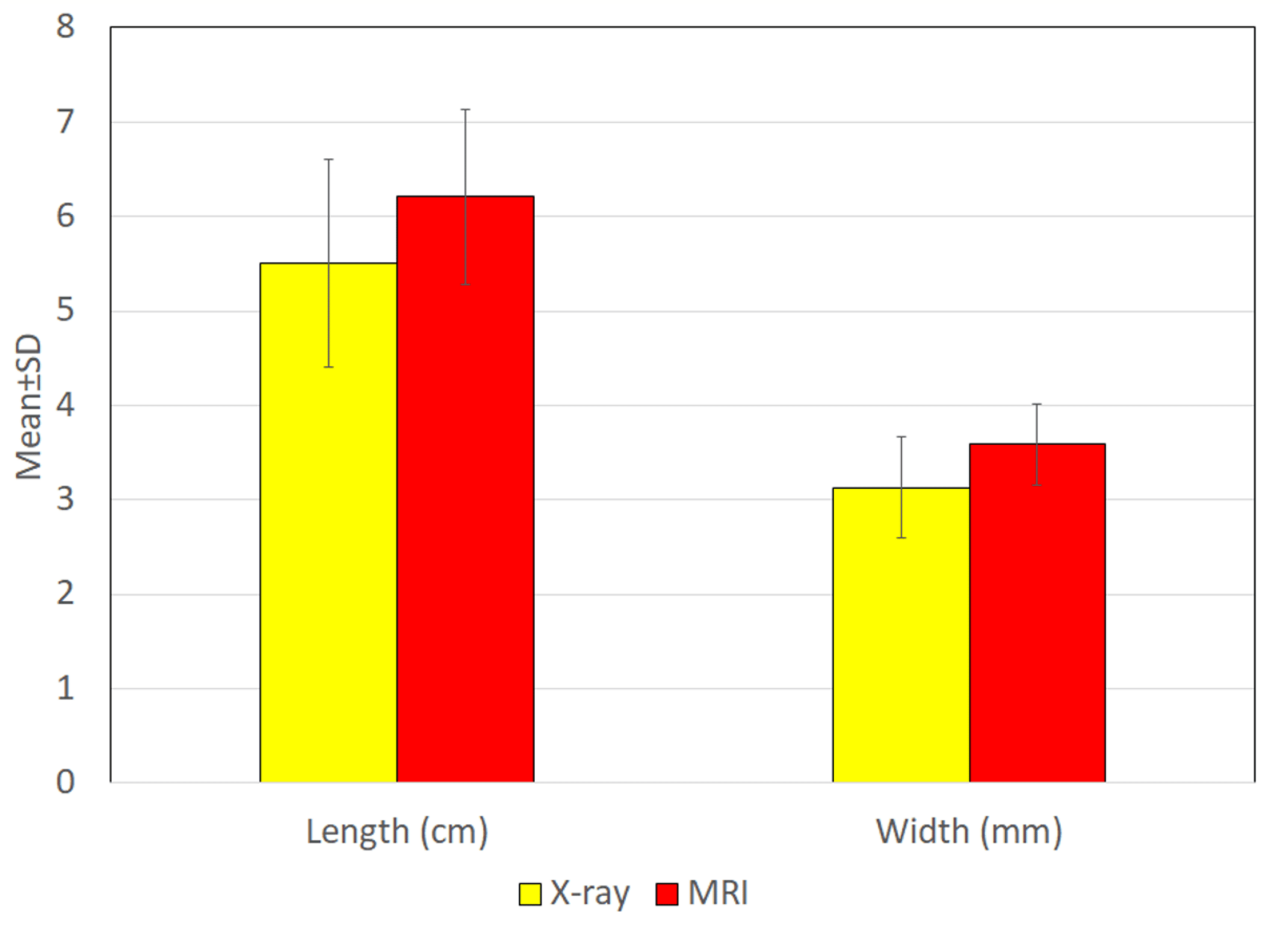
Comparison of dimensions of tract between X-ray and MRI MRI: magnetic resonance imaging

Lesion characteristics as evaluated by X-ray fistulogram (Figure 1) revealed tract lengths ranging from 3.5 to 7.5 cm, with a mean length of 5.50 ± 1.10 cm. The tract widths ranged from 2.0 to 4.4 mm, with a mean width of 3.13 ± 0.53 mm (Table 1). In comparison, MRI demonstrated greater lesion detail, with tract lengths ranging from 4.6 to 8.0 cm (mean 6.21 ± 0.93 cm) and tract widths from 2.0 to 4.4 mm (mean 3.59 ± 0.43 mm) (Table 2). The differences in both length and width measurements between MRI and X-ray fistulogram were statistically significant (p < 0.05) (Figure 2).

**Table 1:** Distribution of study population according to dimensions of lesion as seen on X-ray (n = 52)

SN	Dimensions	No. of cases	Percentage
1.	Length		
	≤5 cm	23	44.2
	5-8 cm	29	55.8
	Mean length ± SD (range) in cm	5.50 ± 1.10 (3.5-7.5)	
2.	Width		
	2-3 mm	20	38.5
	3.1-4 mm	30	57.7
	>4 mm	2	3.8
	Mean width ± SD (range) in mm	3.13 ± 0.53 (2-4.4)	

**Table 2:** Distribution of study population according to dimensions of lesion as seen on MRI (n = 52) MRI: magnetic resonance imaging

SN	Dimensions	No. of cases	Percentage
1.	Length		
	≤5 cm	13	25.0
	5-8 cm	39	75.0
	Mean length ± SD (range) in cm	6.21 ± 0.93 (4.6-8.0)	
2.	Width		
	2-3 mm	2	3.8
	3.1-4 mm	45	86.5
	>4 mm	5	9.6
	Mean width ±SD (range) in mm	3.59 ± 0.43 (2-4.4)	

**Figure 3:**
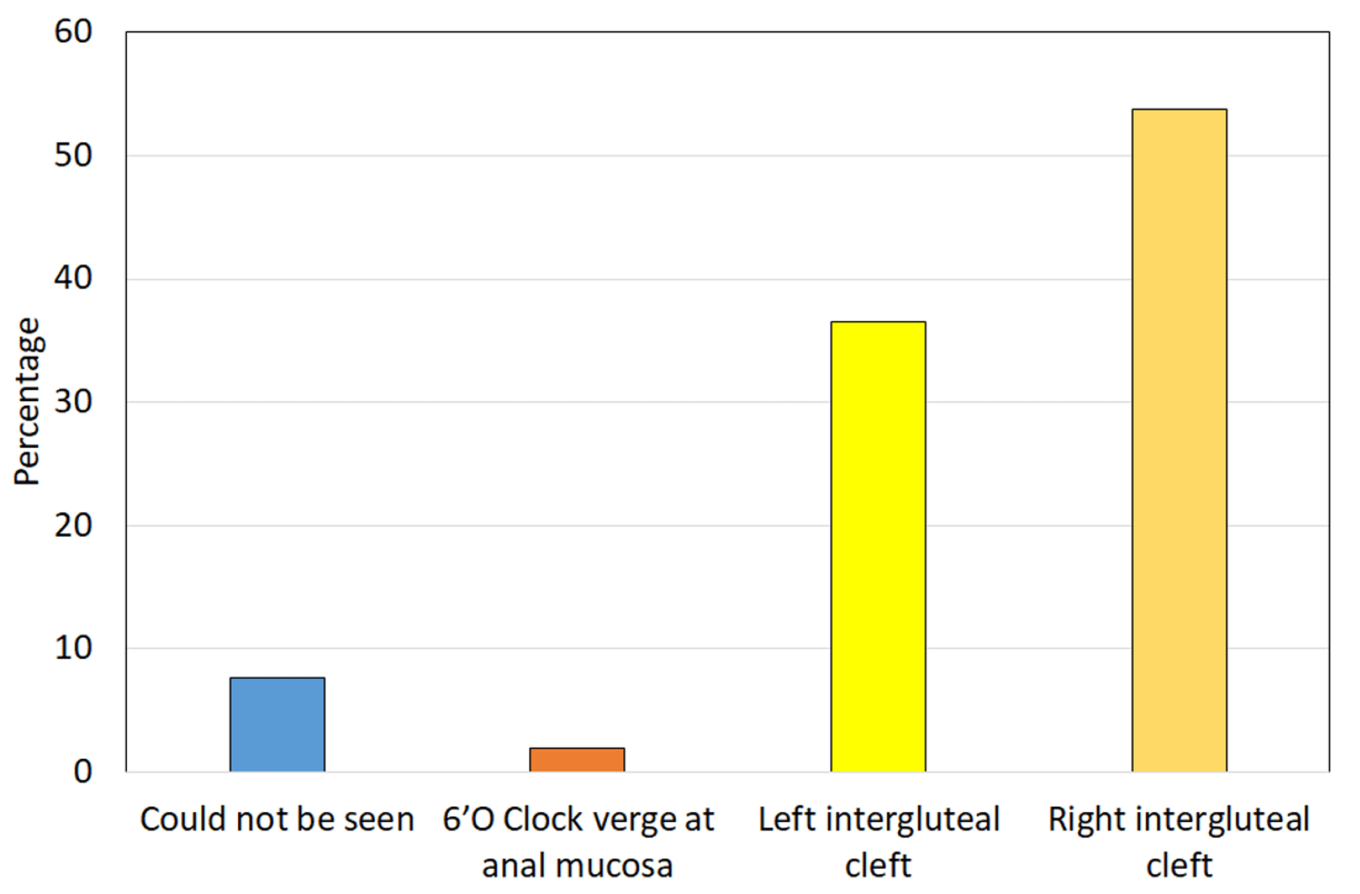
Distribution of study population according to external opening status on MRI MRI: magnetic resonance imaging

**Figure 4:**
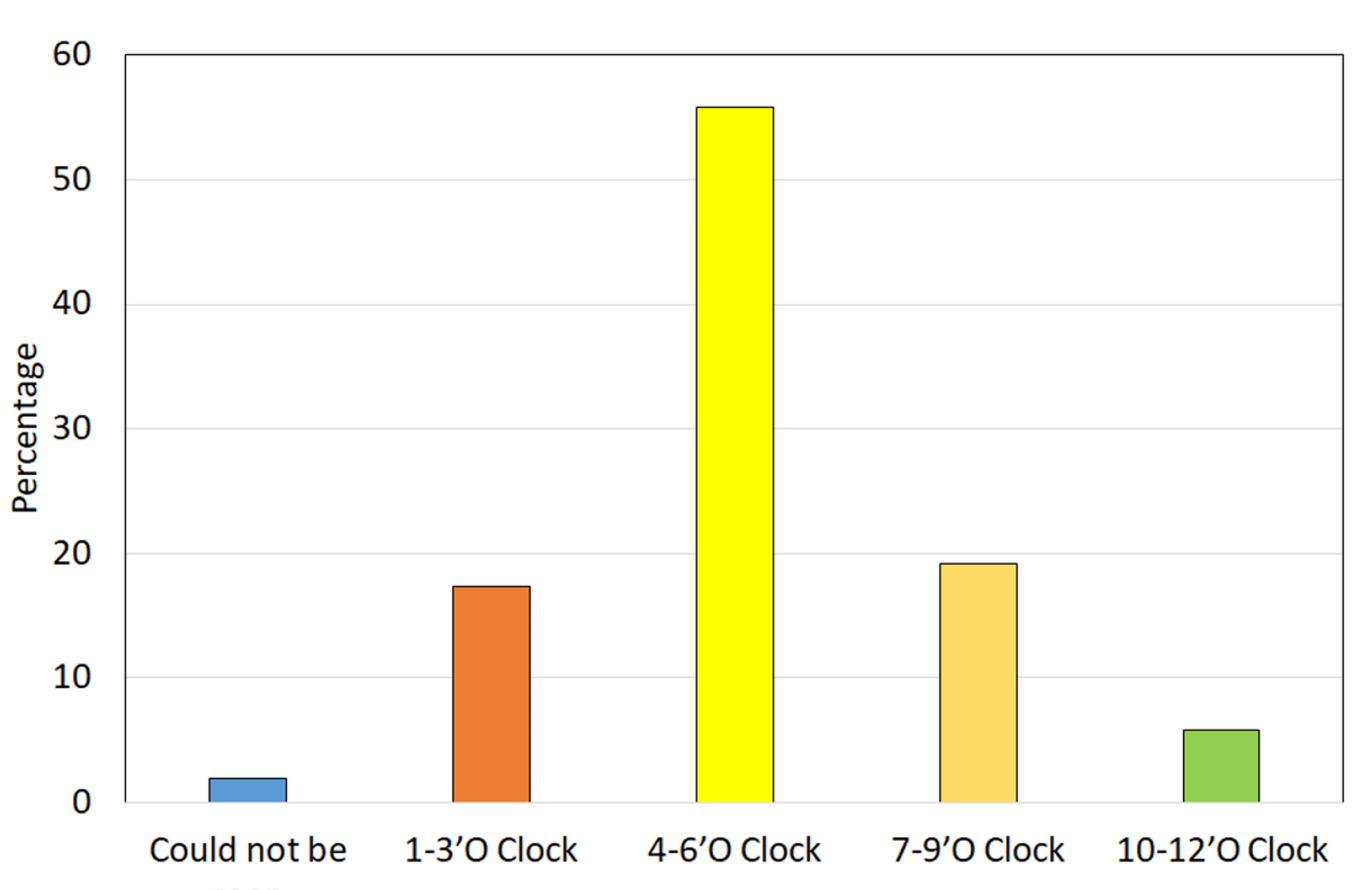
Distribution of study population according to internal opening status on MRI MRI: magnetic resonance imaging

The most frequent location for the external opening was the right intergluteal cleft (53.8%), followed by the left intergluteal cleft (36.5%) (Figure 3). Internal openings were predominantly identified in the four to six o’clock position on MRI (55.8%) (Figure 4). MRI diagnosed the majority of cases as complex fistulas (76.9%), with trans-sphincteric fistulas constituting 13.5% of diagnoses. Other pathologies included sinus tracts (3.8%), hemorrhoids, perianal abscesses with sinus tracts, and pilonidal sinuses (1.9% each).

**Figure 5:**
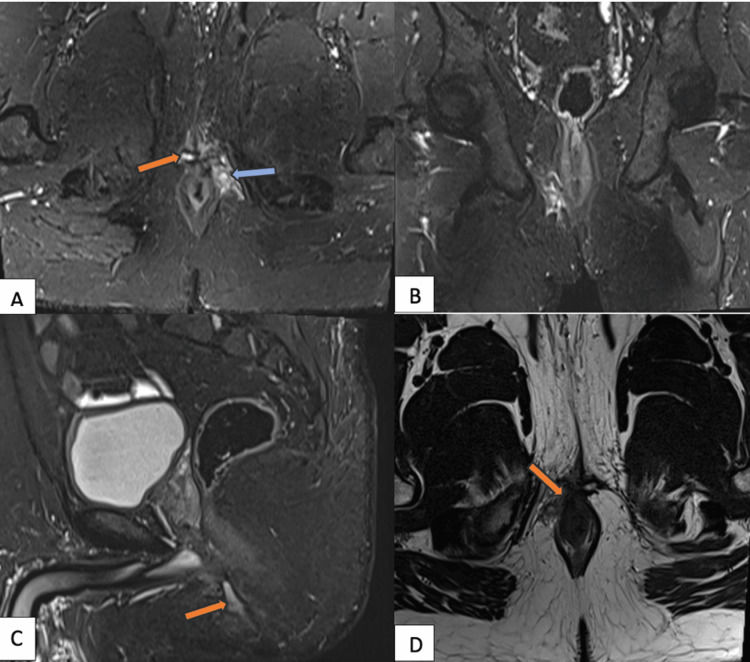
MRI fistulogram demonstrating a St. James grade 4 perianal fistula with secondary ramifications and abscess formation MRI: magnetic resonance imaging; STIR: short tau inversion recovery STIR coronal image (A) shows a transsphincteric fistulous tract (orange arrow) with an associated abscess cavity (blue arrow) in the ischioanal fossa. Coronal (B) and sagittal (C) STIR images reveal multiple ramifications extending into adjacent soft tissues. Axial T2-weighted image (D) further delineates the complex tract traversing both sphincters (orange arrow)

**Figure 6:**
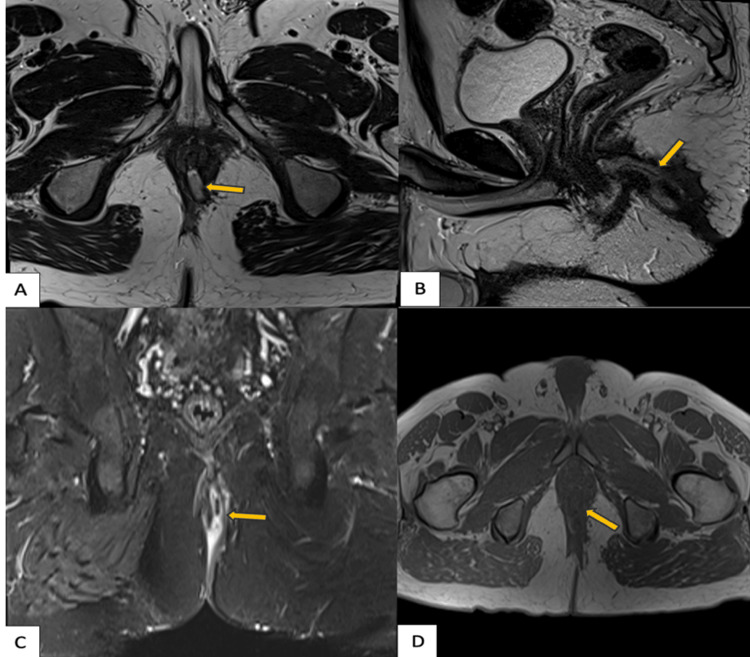
MRI fistulogram demonstrating a St. James grade 3 perianal fistula MRI: magnetic resonance imaging; STIR: short tau inversion recovery Axial (A), sagittal (B), T2-weighted, coronal STIR (C), and axial (D) T1-weighted images reveal a transsphincteric fistulous tract (orange arrows) extending through both the internal and external sphincters into the ischioanal fossa. Mild surrounding inflammation is noted without associated abscess or secondary extensions (C)

**Figure 7:**
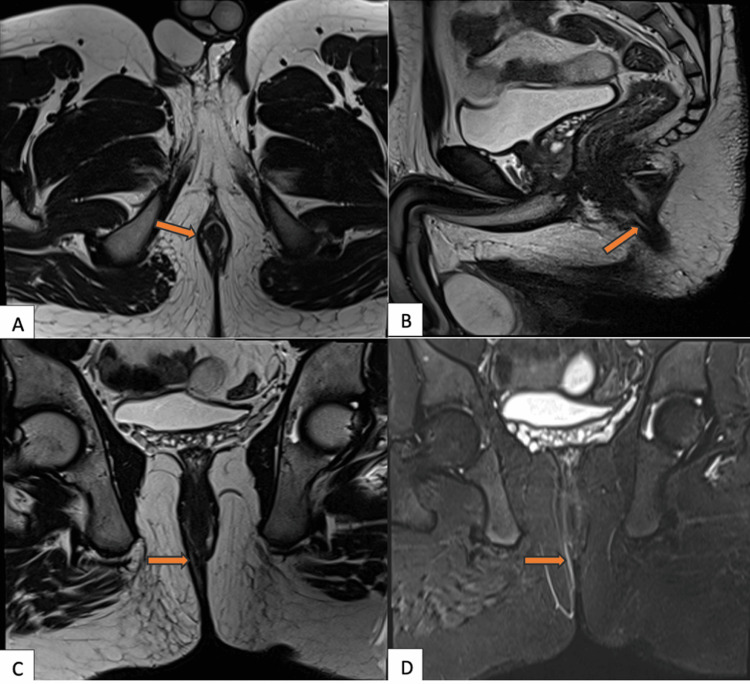
MRI fistulogram demonstrating a St. James grade 1 perianal fistula MRI: magnetic resonance imaging; STIR: short tau inversion recovery Axial (A), sagittal (B), coronal (C) T2-weighted images and coronal STIR (D) image showing a hyperintense linear tract confined to the intersphincteric space (orange arrows) without evidence of abscess or secondary tracts, consistent with a simple intersphincteric fistula

According to the St. James University Hospital MRI classification, the largest proportion of patients were categorized as grade 4 (55.8%) (Figure 5), followed by grade 3 (15.4%) (Figure 6), grade 2 (13.5%), grade 0 (9.6%), grade 1 (3.8%) (Figure 7), and grade 5 (1.9%) (Table 3). Surgical grading was consistent with imaging findings, with grade 4 being the most common (53.8%) (Table 4). MRI findings demonstrated excellent correlation with intraoperative findings in 88.5% of cases, with a statistically significant level of agreement (Cohen’s kappa = 0.820, p < 0.001).

**Table 3:** Distribution of study population according to St. James University Hospital MRI diagnosis (n = 52) MRI: magnetic resonance imaging

SN	Diagnosis	No. of cases	Percentage
1.	Grade 0 (no established fistula tract)	5	9.6
2.	Grade 1 (simple intersphincteric with no abscess or side branch)	2	3.8
3.	Grade 2 (intersphincteric with abscess or side branch)	7	13.5
4.	Grade 3 (transsphincteric with no abscess or side branch)	8	15.4
5.	Grade 4 (transsphincteric with abscess or side branch)	29	55.8
6.	Grade 5 (supralevator or translevator)	1	1.9

**Table 4:** Distribution of study population according to intraoperative grades (n = 52)

SN	Grades	No. of cases	Percentage
1.	Grade 0 (no perianal fistula)	5	9.6
2.	Grade 1	1	1.9
3.	Grade 2	8	15.4
4.	Grade 3	9	17.3
5.	Grade 4	28	53.8
6.	Grade 5	1	1.9

The diagnostic accuracy of MRI was 100% for grades 0 and 5. High levels of accuracy were also observed for grades 1 (98.1%), 2 (94.2%), 3 (90.4%), and 4 (94.2%), underscoring the reliability of MRI in grading and characterizing perianal fistulas (Table 5).

**Table 5:** Diagnostic efficacy of MRI for different St. James grades PPV: positive predictive value; NPV: negative predictive value

SN	St. James grades	Sensitivity	Specificity	PPV	NPV	Accuracy
1.	Grade 0	100%	100%	100%	100%	100%
2.	Grade 1	50%	98.0%	50%	100%	98.1%
3.	Grade 2	75%	95.5%	85.7%	95.6%	94.2%
4.	Grade 3	66.7%	95.7%	75%	93.2%	90.4%
5.	Grade 4	93.1%	95.7%	96.4%	91.7%	94.2%
6.	Grade 5	100%	100%	100%	100%	100%

## Discussion

Preoperative imaging plays a pivotal role in the evaluation and management of perianal fistulas, as it provides essential anatomical detail required for surgical planning and minimizing the risk of recurrence. In the present study, the diagnostic efficacy of X-ray fistulogram was directly compared with that of 3T MRI, with surgical findings used as the reference standard.

This prospective analysis included 52 patients with clinically suspected perianal fistulas. The mean age was 37.40 ± 11.03 years, with most patients (65.4%) falling within the 21-40-year age group. These findings are consistent with those reported by Shahzad et al. and Madany et al., who documented mean ages of 35.2 and 42.4 years, respectively [7,8]. A pronounced male predominance (92.3%) was observed in our cohort, aligning with prior studies that reported male-to-female ratios ranging from 2.4:1 to 29:1. Notably, even in Shahzad et al.’s study, where the ratio was closer to parity, males remained more commonly affected [7].

All patients presented with purulent perianal discharge, and more than half (55.8%) reported associated pain. This clinical profile partially overlaps with that described by Shahzad et al., who noted pain (79.2%), swelling (61.2%), and discharge (56.2%) as prevalent symptoms, with recurrent abscesses present in 25.5% of cases [7].

In comparing imaging modalities, our results demonstrated that X-ray fistulogram significantly underestimated both the length and width of the fistulous tracts compared to MRI. This supports the established limitations of X-ray imaging, which include low soft tissue resolution and poor visualization of complex or high-lying tracts. Furthermore, X-ray fistulogram failed to adequately identify internal openings or sphincter involvement, thereby limiting its clinical relevance in preoperative planning.

In contrast, MRI provided superior anatomical detail across all measured parameters. The mean tract length visualized on MRI was 6.21 ± 0.93 cm, significantly longer than the 5.50 ± 1.10 cm estimated by X-ray, while lesion width was also more accurately delineated by MRI (3.59 ± 0.43 mm vs. 3.13 ± 0.53 mm). These findings echo those of Beets-Tan et al. and Stoker et al., who emphasized the ability of MRI to delineate primary and secondary tracts, internal openings, and associated abscesses with high precision [9,10].

MRI classified the majority of cases (76.9%) as complex fistulas, with over half (55.8%) graded as grade 4 using the St. James University Hospital classification. These results are comparable to those reported by Madany et al. and Kummari et al., who found high-grade fistulas in 38% and 39.6% of their cohorts, respectively [8,11]. While our study identified isolated abscesses and supralevator extension in only 1.9% of cases each, other studies have reported a higher incidence of secondary tracts and abscesses, potentially reflecting variations in patient populations, disease chronicity, or imaging protocols (Table 6).

**Table 6:** Comparison of key findings of the present study with those reported in previously published studies

SN	Author (year)	n	High grade (4 & 5)	Abscess	Supralevator ext.	Horse-shoe ext.	Sec. tract
1.	Mohey and Hassan (2017) [12]	30	6.7%	16.7%	10%	-	-
2.	Madireddy et al. (2020) [13]	40	25%	42.5%	10%	20%	-
3.	Nemade et al. (2020) [14]	50	28%	12%	6%	-	38%
4.	Madany et al. (2023) [8]	50	38%	22%	2%	10%	20%
5.	Kummari et al. (2024) [11]	48	39.6%	-	37.5%	18.8%	33.3%
6.	Shahzad et al. (2025) [7]	98	-	30.6%	-	-	23.5%
7.	Present study (2025)	52	38.5%	1.9%	1.9%	76.9%	-

MRI demonstrated excellent correlation with intraoperative findings, with a diagnostic agreement rate of 88.5% and a statistically significant kappa value (κ = 0.820), indicating almost perfect agreement. The diagnostic accuracy of MRI was particularly high for grades 0 and 5 (100%) and remained above 90% for grades 2 through 4. These findings align with those of Spencer et al. and Bhatt et al., who also reported high sensitivity and specificity of MRI in detecting fistula complexity, internal openings, and associated complications [4,15].

By contrast, X-ray fistulogram contributed little to surgical decision-making, highlighting its obsolescence in modern clinical practice. Its limited ability to evaluate soft tissue structures, poor visualization of complex tracts, and lack of correlation with surgical anatomy underscore the diminished role of this modality in current diagnostic algorithms.

While the findings of this study strongly support the use of 3T MRI in the evaluation of perianal fistulas, certain limitations must be acknowledged. The relatively small sample size may limit the generalizability of results. Additionally, the restricted scope of X-ray evaluation precluded meaningful analysis of associated abscesses or secondary tracts, which may have led to underestimation of its utility in selected cases.

## Conclusions

In conclusion, this study reaffirms the clinical superiority of MRI over X-ray fistulogram in the preoperative assessment of perianal fistulas. Given its high diagnostic accuracy, strong correlation with surgical findings, and ability to delineate complex anatomy, MRI should be regarded as the imaging modality of choice. Future studies with larger sample sizes and comparative assessments involving other imaging techniques, such as endoanal ultrasound, are recommended to further optimize diagnostic strategies.

This study establishes 3T MRI fistulogram as a highly accurate and superior modality for the preoperative evaluation of perianal fistulas, demonstrating excellent correlation with intraoperative findings and significantly outperforming conventional X-ray fistulogram in delineating tract anatomy, internal and external openings, and complex extensions. With high sensitivity and specificity, especially for advanced fistula grades, MRI enables precise classification and surgical planning, reinforcing its role as the imaging gold standard, while X-ray fistulography offers limited utility and may be reserved for settings where MRI is unavailable.
